# OCaPPI-Db: an oligonucleotide probe database for pathogen identification through hybridization capture

**DOI:** 10.1093/database/baw172

**Published:** 2017-02-26

**Authors:** Cyrielle Gasc, Antony Constantin, Faouzi Jaziri, Pierre Peyret

**Affiliations:** 1 Université Clermont Auvergne, INRA, MEDIS, F-63000 Clermont-Ferrand, France; 2 Université d’Auvergne, IUT du Puy en Velay, Le Puy en Velay, France; 3 Université d’Auvergne, ISIT, Clermont-Ferrand, France

## Abstract

The detection and identification of bacterial pathogens involved in acts of bio- and agroterrorism are essential to avoid pathogen dispersal in the environment and propagation within the population. Conventional molecular methods, such as PCR amplification, DNA microarrays or shotgun sequencing, are subject to various limitations when assessing environmental samples, which can lead to inaccurate findings. We developed a hybridization capture strategy that uses a set of oligonucleotide probes to target and enrich biomarkers of interest in environmental samples. Here, we present Oligonucleotide Capture Probes for Pathogen Identification Database (OCaPPI-Db), an online capture probe database containing a set of 1,685 oligonucleotide probes allowing for the detection and identification of 30 biothreat agents up to the species level. This probe set can be used in its entirety as a comprehensive diagnostic tool or can be restricted to a set of probes targeting a specific pathogen or virulence factor according to the user’s needs.

**Database URL**: http://ocappidb.uca.works

## Introduction

Bacterial pathogens are a major public health concern because of their ability to cause severe illness. Some bacteria, including *Bacillus anthracis*, *Yersinia pestis* and *Francisella tularensis*, are prone to be used in acts of bio- and agroterrorism due to their ease of dissemination, low infective doses and high morbidity rates (1, 2). Therefore, pathogen species belonging to 26 bacterial genera have been classified in the pathogen priority list of the National Institute of Allergy and Infectious Diseases (NIAID), which is composed of organisms that pose the highest risk to public security (https://www.niaid.nih.gov/). These pathogens are subclassified into categories A, B and C according to their virulence and ease of dissemination.

To minimize the consequences of the intentional, accidental or natural release of these pathogens into the environment and to avoid propagation of these pathogens within the population, technologies that can rapidly and accurately detect and identify biological agents are required. The environmental context of the dissemination of these pathogens needs to be taken into account when designing detection strategies. Indeed, methods based on culture and biochemical tests are valuable for clinical samples, but not for environmental samples because of their low sensitivity and their inability to directly detect the microorganisms present at or below the risk levels from these complex samples (3). Culture-independent molecular methods are more suitable for environmental diagnostics (4). Classical and real-time polymerase chain reaction (PCR) approaches are highly specific and sensitive for pathogen detection, but because of the short length of amplified fragments, these methods only provide information on the presence/absence of the pathogen and their use is limited for high-resolution identification and studies of molecular epidemiology. DNA microarrays (5) can provide information on different pathogenic microorganisms or different biomarkers of one pathogen simultaneously, which is advantageous for environmental samples. Nevertheless, microarrays often require a pre-hybridization PCR amplification step and are subject to false positive or negative rates, which are highly problematic when dealing with deadly biological pathogens. Finally, shotgun sequencing of environmental samples can detect known, unknown, unsuspected and even emerging pathogens, but only if present in high quantities, which is rarely the case for such biological agents (6).

As an alternative to traditional methods, we developed a hybridization capture strategy that allows for the enrichment of DNA sequences containing targeted biomarkers from metagenomics samples through the use of target-specific oligonucleotide probes (7, 8). We demonstrated that after enrichment, the probe-targeted biomarker can represent >40% of the obtained sequences compared with shotgun sequencing in which only 0.003% of the sequences corresponded to the targeted gene (9). This hybridization capture method is able to detect the targeted biomarker and its unknown variants even if present in very low copy numbers in complex environments (9–11). Indeed, thanks to the enrichment and the sensitivity of the method, we were able to reveal the presence of extremely rare biomarkers that could not be directly detected by quantitative PCR. The approach also permits to enrich the complete biomarker and its flanking regions over tens of kilobase pairs (kbp), allowing for precise identification at the species or strain level. To optimize throughput, hybridization capture can be used for the simultaneous analysis of one to several hundreds of biomarkers in several samples simultaneously.

Here, we present the Oligonucleotide Capture Probes for Pathogen Identification Database (OCaPPI-Db), which is an oligonucleotide hybridization capture probe database targeting virulence factors and genes used to discriminate the NIAID’s priority list for bacterial pathogens. The use of this probe set coupled with a hybridization capture strategy could be used as a sensitive and efficient alternative diagnostic tool to detect and identify bioterrorism agents from various environmental samples to prevent propagation of these agents within the population.

## Database construction and development

### Sequence collection

**Figure 1.**
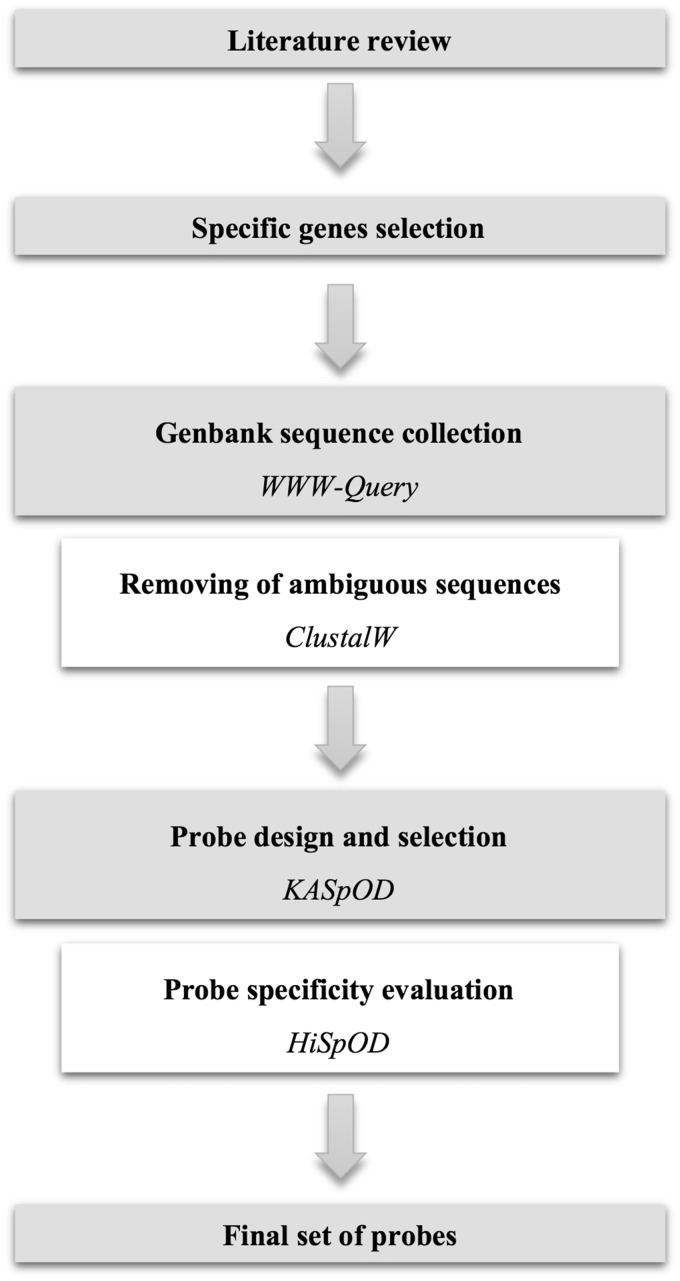
Overview of the database construction workflow.

To target the NIAID’s 30 priority bacterial genera or species through hybridization capture (Table 1), previously published studies were reviewed to obtain information regarding biomarkers used to detect and identify each of the pathogens. Specific genes from existing databases of pathogen virulence factors such as VFDB (12) were also utilized to complete the list of biomarkers. Finally, a list of specific biomarkers was defined for each of the bacteria (Figure 1). These biomarkers can be virulence factors, broadly defined, functional genes, antibiotic resistance genes or any other genes allowing for the specific enrichment of the pathogen at the genus level, and whenever possible at the species level. If no genes were species-specific, biomarkers were selected so that all, or a subsection, of the species from the corresponding genus are enriched in a manner where species can be discriminated based on small biomarker sequence variations. A total of 245 biomarkers were thus selected to target the 30 bacterial genera or species of interest (Table 1).

**Table 1.** Database composition

Category	Bacterial pathogens	Number of targeted biomarkers	Number of probes designed
A	*Bacillus anthracis*	14	126
	*Clostridium botulinum*	14	185
	*Francisella tularensis*	11	84
	*Yersinia pestis*	12	86
B	*Brucella* species	9	38
	*Burkholderia* species	5	19
	*Campylobacter jejuni*	12	65
	*Chlamydiophyila psittaci*	4	25
	*Coxiella burnetii*	6	38
	*Escherichia coli*	19	121
	*Clostridium perfringens*	8	66
	*Listeria monocytogenes*	17	124
	*Vibrio* species	8	46
	*Salmonella* species	8	58
	*Shigella* species	8	38
	*Staphylococcus aureus*	12	57
	*Rickettsia prowazekii*	2	33
	*Yersinia enterocolitica*	12	53
C	*Rickettsia* species	3	28
	*Mycobacterium tuberculosis*	9	55
C (Emerging)	*Anaplasma phagocytophilum*	3	29
	*Bartonella henselae*	5	35
	*Bordetella pertussis*	6	65
	*Borrelia burgdorferi*	3	14
	*Borrelia miyamotoi*	3	16
	*Clostridium difficile*	3	26
	*Ehrlichia* species	5	17
	*Enterococcus* species	8	65
	*Leptospira* species	6	32
	*Streptococcus pyogenes*	10	41

Fasta sequences of the selected genes were collected from GenBank using the WWW-Query research tool (http://doua.prabi.fr/search/query_fam.php) with the names of the organism and the gene targeted as keywords (Figure 1). Collected sequences for each gene were then aligned using ClustalW (13) to visually ensure that all sequences correspond to the gene of interest and to remove from the sequence data set any sequences arising from erroneous gene annotation (Figure 1).

### Probe design

Probes were designed using KASpOD (K-mer based Algorithm for high-Specific Oligonucleotide Design) software (14) (Figure 1). KASpOD is a fast k-mer-based software that allows for the selection of highly specific and explorative probes based on large sequence data sets. KASpOD first extracts group-specific k-mers from targeted and non-targeted datasets if provided, and removes k-mers found in both groups to keep only targeted data set-specific probes. Fully overlapping k-mers are then clustered using CD-HIT (15) at an 88% identity threshold. For each cluster, a degenerate consensus probe is constructed taking into account sequence variability at each position. Coverage of each degenerate consensus probe specific for the targeted data set is then measured using PatMaN (16) and specificity is assessed in the same way by comparing degenerate probes against the non-target group sequences.

Pathogen-targeting probe design was conducted using the selected gene sequences, and non-targeted gene sequences if necessary, to obtain genus- or species-specific probes. The probe length was set to 80-mer, and the edit distance, defined as the total number of differences, gaps and/or mismatches allowed between the probes and the full-length sequences, was set to 3. Among all the degenerate probes designed for each gene, a minimal set of probes was selected so that probes are distributed uniformly over the full-length gene, with approximately one probe for each 200 bp and no probe overlap (Figure 1). Probes with the best coverage were preferentially selected to target the largest gene diversity, and probes showing cross-hybridizations with non-targeted sequences were excluded.

### Probe validation

The quality and specificity of each probe were evaluated using HiSpoD (High Specific Oligo Design) (17) (Figure 1), a functional microarray probe design algorithm that uses individual nucleic acid sequences or consensus degenerate sequences to design highly specific probes. For each sequence, the algorithm selects all probes of a given size along the sequence by incrementing the constant defined probe size in a window. Probes are then filtered according to their degeneracy, melting temperature (*T*_m_) and complexity stretch (maximum authorized number of identical consecutive bases). Probes that successfully pass the first step are then assessed for similarity using the BLASTN program against the dedicated EnvExBase database containing approximately 10 million microbial coding data sequences (CDSs) from the EMBL databank. Percent identity and similarity stretch are then determined for all positive results from the previous BLASTN analysis to identify potential cross-hybridizations. The algorithm finally provides all filtered oligonucleotides, the list of sequences targeted by each probe and their potential cross-hybridizations.

Thereby, all the probes selected for each gene were validated for quality and specificity using HiSpOD. The probe length was set to 80-mer so that probes were directly subjected to filtering and specificity tests without any other probe determination step. The Tm range was designated between 50 and 80 °C, the maximal degeneracy threshold was set to 512 and the complexity stretch was set to 10. A similarity search was performed with a 90% similarity threshold between the probe and non-target sequences and a 15-mer maximal consecutive match of probes with the non-target sequences. Probes meeting the quality criteria and specific for the targeted gene and genus were selected for the hybridization capture probe set. A total of 1,685 probes targeting 245 genes from the NIAID’s 30 priority bacterial genera or species were thus obtained (Table 1).

### Web interface

**Figure 2.**
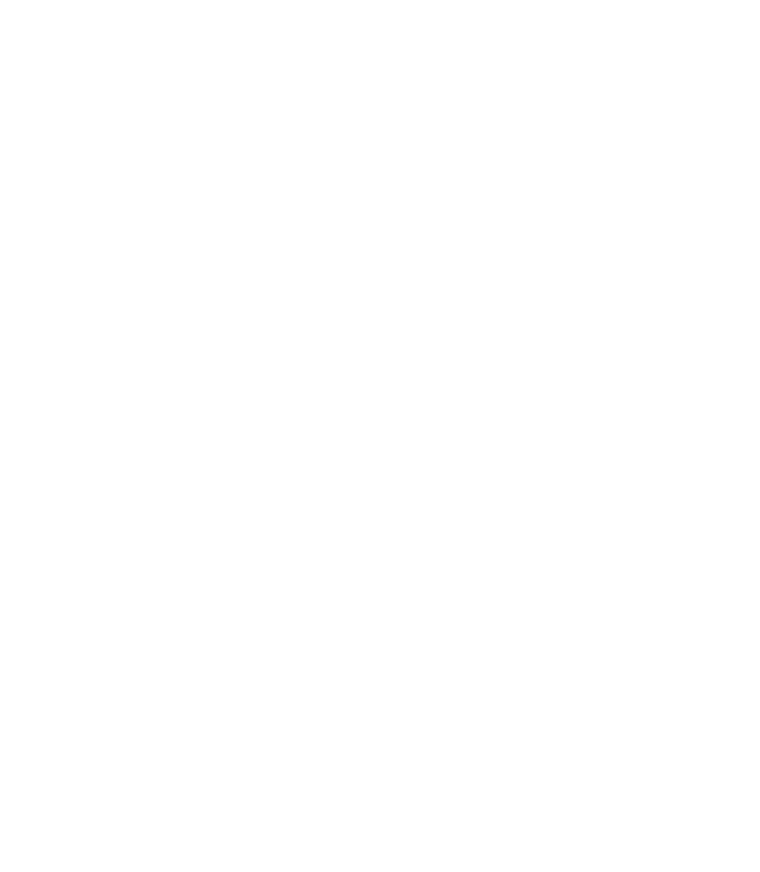
Screenshots of the OCaPPI-Db web interface.

To make all the pathogen-targeting probes easily available, we developed a web interface to freely access and download the 1,685 probes that compose our database (Figure 2). The website, freely available online at http://ocappidb.uca.works, was implemented using Framework Symfony 3 (PHP 7), ORM Doctrine 2 (MySQL 5) and HTML 5/CSS 3/java-script for UI. Based on the NIAID’s pathogen categories A, B and C, the OCaPPI-Db web interface provides a hierarchical browse of the database content. When a pathogen is selected, the probes designed for all the genes of this genus or species are then displayed. Associated information is provided for each probe: category, phylogenetic assignment of the pathogen, gene name, gene function, gene length, gene IDs, probe ID, probe sequence, probe length, probe degeneracy, start and end position, *T*_m_, GC%, gene sequences coverage, sequences covered and design tool. Probes can also be obtained using a rapid search with keywords for a genus or a biomarker name, a gene function or any of the information provided for the probes. Only probes that match these keywords are then displayed. Probes can also be obtained through an advanced search using multiple criteria. Selected probes can be downloaded both in tabular and FASTA formats. The gene sequences used to design each probe can also be downloaded through the download tool.

## Discussion

The detection and identification of bacterial pathogens involved in acts of bio- and agroterrorism is essential to avoid their dispersal in the environment and their propagation within the population. Conventional molecular methods, such as PCR amplification, DNA microarrays or shotgun sequencing, are better for identifying pathogens in environmental samples compared to culture or biochemical methods. Nevertheless, these available molecular methods are limited in accuracy and sensitivity when assessing complex environmental samples (4).

As an alternative method, we developed a hybridization capture strategy that uses a set of oligonucleotide probes to target and enrich biomarkers of interest and their potential unknown variants in environmental samples (7). Due to our method’s specificity, sensitivity and ability to simultaneously assess several biomarkers, this strategy appears well suited for the detection and identification of agents of bio- and agroterrorism in environmental samples. Therefore, we constructed OCaPPI-Db, a capture probe database containing a set of 1,685 80-mer oligonucleotide probes allowing for the specific enrichment of the NIAID’s 30 category A, B and C bacterial genera or species (Table 1). These probes target 245 different biomarkers correspond to virulence factors but also any other genes allowing for the specific enrichment of the pathogens of interest.

This probe set can be used in its entirety in hybridization capture experiments to simultaneously detect and identify all the above-mentioned pathogens with potential for use as agents of bio- or agroterrorism due to the enrichment of their associated biomarkers. Alternatively, probes targeting genes belonging to a particular genus can be selected to obtain a genus-specific diagnostic tool; probes targeting toxin-coding genes from all pathogens can be selected to evaluate the potential pathogenicity of the present species; or probes targeting one biomarker, specific for one bacterial genus, can be selected to enrich this specific gene and to identify gene variants potentially responsible for enhancement of a pathogen’s virulence. Thus, a desired set of probes can be selected from the complete database to adapt this scalable tool to the diagnostic requirements.

Probe design has been performed so that probes are gene-specific, and at least genus-specific. Nevertheless, based on selected genus biomarker sequences or a combination of detected genes, species discrimination is possible. Moreover, due to the enrichment of complete biomarkers and their flanking regions over several tens of kbp by hybridization capture, reconstruction of very long subsets of genomes or even complete genomes is possible. Based on this sequence data, identification of pathogens can reach the strain- or biovar-level and provide insight regarding organism origin and evolution. Furthermore, due to the explorative design of probes, all variants of the targeted biomarkers are captured, allowing for the possibility of detecting all pathogens, including modified and unknown bacterial agents. This information obtained from hybridization capture can be used to confirm the information provided by cultivation methods or even help the isolation of pathogens for further studies.

Future work will be directed towards the application of hybridization capture to environmental samples contaminated with biothreat agents with the complete or partial set of probes to determine the sensibility and specificity of our method, and towards the characterization of the benefits of using our strategy compared to conventional molecular methods. Nevertheless, given the sensitivity and specificity of the probes designed with the same strategy and used in different hybridization capture experiments on metagenomic samples (9–11), the efficiency of the probes listed in OCaPPI-Db should be confirmed. Other sets of probes targeting the viral and fungal pathogens of the NIAID’s priority list will be designed to provide a comprehensive diagnostic tool capable of detecting and identifying any biothreat agent. Additionally, the enrichment of the database with probes targeting pathogens of clinical importance or targeting an exhaustive list of antibiotic resistance genes may also be a future endeavor. OCaPPI-Db will be updated annually adding newly designed probes and re-evaluating probe specificity considering the new sequences published in the databases.
